# Weak Genetic Structure in Northern African Dromedary Camels Reflects Their Unique Evolutionary History

**DOI:** 10.1371/journal.pone.0168672

**Published:** 2017-01-19

**Authors:** Youcef Amine Cherifi, Suheil Bechir Semir Gaouar, Rosangela Guastamacchia, Khalid Ahmed El-Bahrawy, Asmaa Mohammed Aly Abushady, Abdoallah Aboelnasr Sharaf, Derradji Harek, Giovanni Michele Lacalandra, Nadhira Saïdi-Mehtar, Elena Ciani

**Affiliations:** 1 Laboratory of Molecular and Cellular Genetics, Department of Applied Molecular Genetics, University of Science and Technology of Oran “Mohamed BOUDIAF” (USTOMB), El Mnaouar, BP, Bir El Djir, Oran, Algeria; 2 Department of Biology, Aboubakr Belkaid Tlemcen University, 22 Rue Abi Ayed Abdelkrim Fg Pasteur B.P, Tlemcen, Algeria; 3 Department of Biosciences, Biotechnologies, Biopharmaceutics, University of Bari “Aldo Moro”, Bari, Italy; 4 Department of Emergency and Organ Transplatation, Section of Veterinary Clinics and Animal Productions, University of Bari “Aldo Moro”, SP per Casamassima, Valenzano, Bari, Italy; 5 Animal & Poultry Production Division, Desert Research Center (DRC), El Matariya, Cairo, Egypt; 6 Department of Genetics, Faculty of Agriculture, Ain Shams University, Cairo, Egypt; 7 Institut National de la Recherche Agronomique, 2 rue les frères OUADEK-BP N 200 Hassen Badi EL-Harrach Alger, Algeria; Embrapa, BRAZIL

## Abstract

Knowledge on genetic diversity and structure of camel populations is fundamental for sustainable herd management and breeding program implementation in this species. Here we characterized a total of 331 camels from Northern Africa, representative of six populations and thirteen Algerian and Egyptian geographic regions, using 20 STR markers. The nineteen polymorphic loci displayed an average of 9.79 ± 5.31 alleles, ranging from 2 (CVRL8) to 24 (CVRL1D). Average H_e_ was 0.647 ± 0.173. Eleven loci deviated significantly from Hardy-Weinberg proportions (P<0.05), due to excess of homozygous genotypes in all cases except one (CMS18). Distribution of genetic diversity along a weak geographic gradient as suggested by network analysis was not supported by either unsupervised and supervised Bayesian clustering. Traditional extensive/nomadic herding practices, together with the historical use as a long-range beast of burden and its peculiar evolutionary history, with domestication likely occurring from a bottlenecked and geographically confined wild progenitor, may explain the observed genetic patterns.

## Introduction

The one-humped dromedary camel (*Camelus dromedarius*) belongs, together with the two-humped *C*. *bactrianus* and *C*. *ferus* species, to the Old-World camelids. It is generally accepted to be among the last major livestock species to have been domesticated. Domestication of dromedaries is acknowledged to have occurred in Asia, sometime not before the third millennium BCE. Despite several studies have addressed this issue [1–21], when and where the dromedary was actually domesticated is still a matter of research. Recently, Almathen et al. (2016), by using genotypic data from microsatellites and mitochondrial modern and ancient DNA, described the genetic basis of dromedary domestication, as well as the Southeast coast of the Arabian Peninsula as one possible place of origin. The first domesticated dromedary camels were probably mainly used as a source of meat and/or milk, and only later they significantly contributed, as pack animals, to the development of long distance commercial routes [12,22].

Introduction of dromedary into Africa is thought to have occurred through (i) an early sea-route (middle of the 1^st^ millennium BCE; Epstein 1971) into Somalia, and (ii) a land-route (dates ranging 3^rd^ millennium BCE to 7^th^ century BCE) into transcontinental Egypt. Here, the Semitic word for “dromedary” is attested for the first time in a papyrus from the XXV dynasty, in a period corresponding to the Assyrian invasion of Egypt [23]. Since then, dromedary bone remains are increasingly found in Egypt, but it is only in Roman times that dromedary dispersal into Northern Africa, westward of Egypt, occurred [24].

With the end of traditional trans-Saharan caravan routes, the dromedary has lost its central role as ship of the desert. Notwithstanding, it is still used in transport, draught and agriculture activities in several harsh regions in Africa and Asia, significantly contributing to economical and food security of local pastoralist communities [25]. In the last years, the camel farming system has experienced rapid changes, with increasing set-up of periurban dairy farms and dairy plants, diversification of camel products and market penetration [26]. This trend toward a more intensive production system is accompanied by increasing use of artificial insemination, higher culling rates, and higher selection pressure on the best performing animals. These practices may significantly influence genetic structure and variability of the dromedary camel populations in the near future. A characterization of the current levels of diversity is therefore urgent for those populations where this information is partial or lacking.

Overall, Egypt and Algeria count 486 thousand dromedaries [27] which represent an important economic and cultural resource for arid and semi-arid areas. Four camel populations, Sudani, Maghrabi, Falahi (or Fellahi, or Baladi) and Mowalled, are generally recognized in Egypt [28]. On the contrary, several camel populations have been suggested to exist in Algeria based on phenotypic traits [29]. More recently, Cherifi et al [30]., who performed a wide phenotypic and ethno-geographical survey of camels in South-Western Algeria, pointed to the lack of standardization in camel classification criteria, and to the presence of different camel types mixed within single herds. Based on a socio-geographical criterion, they identified the following populations in South-Western Algeria: Targui, characterized by clear coat colour, pronounced muscularity, and appreciated for racing; Rguibi, a multi-purpose well-adapted animal, characterized by various size variants and medium-length hairs; Azawad, slender-looking, light-colored, and hairless, also suitable for race; the small Steppe camel, currently in numerical decline, very appreciated for the quality of hair, especially for producing traditional items; the Ouled Sidi Cheikh camel, well adapted to the dry summer and the very cold winter of the highlands; the Sahraoui camel, obtained by crossing Ouled Sidi Cheikh with the Chaambi population. However, no genetic study has been yet carried out to support phenotypic and socio-geographical classification criteria. Indeed, a considerable amount of gene-flow and admixture is known to exist among different dromedary populations within Algeria and even at a cross-border level, mainly with Mali and Niger (southward), and Maroc (westward) [29]. As knowledge on regional genetic diversity and structure is fundamental for sustainable herd management and implementation of breeding programs in this species, we investigated genetic diversity and relationships among Northern African dromedary camels using multi-allelic STR (Short Tandem Repeat, or microsatellite) markers, a tool that has been extensively shown to be useful for gaining insights into the genetic structure of livestock species [31]. No ultimate clue of population originality and separateness being available for the considered Algerian and Egyptian dromedary populations, we decided to analyze genetic relationships in our dataset arranged both (i) into six populations and (ii) into thirteen geographic regions.

## Materials and Methods

### Sampling

**Fig 1:**
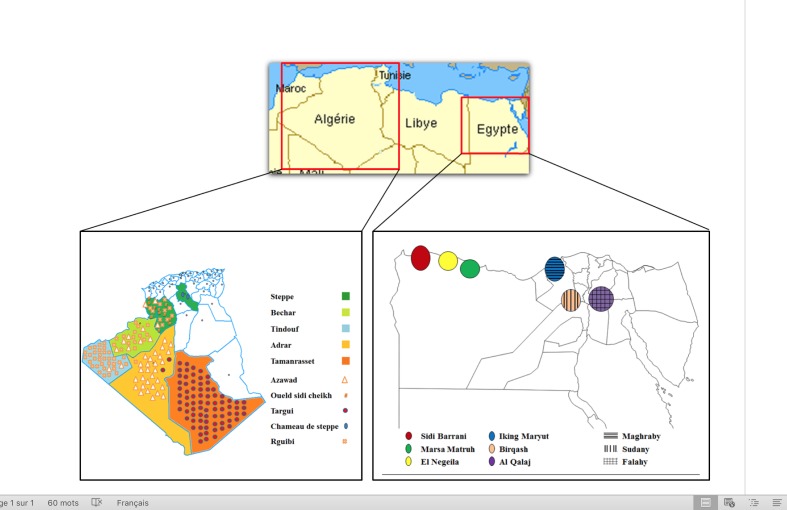
Geographic distribution of the Algerian and Egyptian dromedary samples analyzed in the study.

Blood samples from 198 unrelated animals were collected in EDTA tubes from seven Algerian areas (Naama, n = 5; El Bayadh, n = 17; Djelfa, n = 2; Bechar, n = 22; Tindouf, n = 34; Adrar, n = 31; Tamanrasset, n = 87) representing 79 herds and 17 municipalities distributed almost all over the entire country (Fig 1). Blood samples were also collected from 133 animals sampled in 6 Egyptian areas: Sidi Barrani (n = 24), Negeila (n = 23), Marsa Matruh (n = 16), Iking Maryut (n = 30), Birqash (n = 17), Al Qalaj (n = 23) (Fig 1). The blood used for all of the analyses was collected by veterinarians during routine blood sampling on commercial farm animals. Those animals were not linked to any experimental design and blood sampling was not performed specifically for this study, therefore no ethical authorization was required. All the samples and data processed in our study were obtained with the breeders and breeding organizations' consent. All the samples were collected in close collaboration with the Algerian Directorate of Agriculture Service (DSA), thus in perfect conformity with its ethical guidelines.The majority of Algerian samples (n = 185) belonged to one of the three main Algerian populations, named after the name of the tribe from which the herd’s owner originated (Azawad, Rguibi, Targui), the few remaining samples belonging to the endangered Chameau de steppe (n = 2), the Oueld Sidi Cheikh (n = 2) or population crosses (n = 9) (S1 Table). These thirteen animals were omitted when data arranged into different populations were analyzed. Out of the 133 Egyptian samples, 70 (those from Iking Maryut, Birqash, and Al Qalaj) belonged to one of the three main camel populations (Maghraby, Sudany, and Falahy, respectively), the remaining 63 (from Sidi Barrani, Negeila and Marsa Matruh) being of unknown breed origin (S1 Table). The latter were omitted when data arranged into different populations were analyzed. Genomic DNA was isolated from whole blood using the DNA Extraction Kit (Stratagene, USA) following manufacturer’s instructions.

### STR analysis

Twenty STR loci, out of which 18 belonged to the recommended ISAG/FAO panel [32], were selected (S2 Table). Amplification of STR loci was carried out adopting three previously developed multiplex PCR reactions [33]. PCR products were detected and discriminated by capillary electrophoresis (CE) using an ABI PRISM 310 Genetic Analyzer (Applied Biosystems); 0.5 μl of GeneScan 500 ROX (Life Technologies) were used as internal size standard. Raw CE data were analyzed using the GeneMapper software (Applied Biosystems). In order to avoid issues related with allele size standardization, genotypes were all produced in the same laboratory (Department of Biosciences, Biotechnologies, Biopharmaceutics—Bari, Italy), under the supervision of the same analyst (Rosangela Guastamacchia), and including three reference samples in any CE run. Genotypes are available as Supporting Information (S1 Dataset).

### Statistical analysis

Classical population genetic parameters (allelic frequencies, number of alleles per locus, observed and expected heterozygosity), Hardy-Weinberg (HW) equilibrium, gametic unbalance, genetic differentiation (*F*_ST_) were calculated using the Arlequin software package [34]. Allelic richness and private allelic richness were estimated using HP-RARE [35] adopting n = 15 (30 “genes”) as rarefied samples size. The genetic structure of the populations was analysed using the unsupervised Bayesian clustering algorithms implemented in the software STRUCTURE 2.[36]. STRUCTURE HARVESTER [37] was adopted to visualize STRUCTURE outputs and estimate Evanno’s DeltaK statistics [38]. To test for consistency among runs, each K value (number of assumed clusters in the population) from 1 to 12 was tested 30 times. Relationships among breeds were also explored by Neighbor network analysis using the distance of Reynolds *et al*. (1983)[39]and Nei (1983) calculated by Power Marker [40]. Network representations were adopted as they allow accounting for gene flow among breeds (reticulation) and thus providing a more plausible reconstruction than linear tree representations. Neighbor networks were constructed using the Neighbor-Net algorithm [41] implemented in the SplitsTree4 package v. 4.13.1 [42]. To support inference from network topologies, neighbor-joining (NJ) trees were also generated using the distance of Reynolds *et al*. (1983)[39]and Nei (1983), and adopting 1000 bootstrap replications. Finally, to exclude potential population structure due to relatedness, we used COANCESTRY v.1.0.1.5 [43]. Notably, we adopted the triadic likelihood estimator of pair-wise relatedness (TrioML), which allows for inbreeding and accounts for genotype errors in data. Proportions of pair-wise comparisons falling in different relatedness classes (R ≤ 0.25, Unrelated; 0.25 < R ≤0.5, Half-siblings; R > 0.5, Full-siblings) were calculated.

## Results

### Genetic variability in the total sample and by geographical area

In this study we investigated the genetic variability of Algerian and Egyptian dromedary camels from thirteen geographical areas (Fig 1). Only few samples were collected in the central region of Algeria (Naama, El Bayadh and Djelfa) due to the presence in this area of only a limited number of herds. As a consequence, we carried out the subsequent analyses considering samples from Naama, El Bayadh and Djelfa as coming from a single region, that we labelled as “Steppe”.

All the considered loci were polymorphic (S3 Table), with the exception of CMS17 (data not shown). The locus VOLP32 was the most informative (highest F_ST_ value, 0.07). The number of alleles in the total sample ranged from 2 (CVRL8) to 24 (CVRL1D), with an average value of 9.79 ±5.31. The above loci generally displayed the lowest and the highest number of alleles also within the five Algerian and the six Egyptian regions (S4 Table). Since sample sizes varied among regions, we calculated allelic richness (S1 and S4 Tables) No remarkable difference was observed between regions in allelic richness values, that ranged from 5.1 (Marsa Matruh) to 5.9 (Adrar). Private allelic richness ranged from 0.05 (Iking Maryut) to 0.22 (Adrar) (data not shown). Average observed heterozygosity in the total sample was 0.611 ± 0.169, while gene diversity averaged over loci was 0.647 ± 0.173 (S3 Table). The highest gene diversity was observed in the Egyptian sample from Birqash (0.66 ± 0.15), the lowest being observed in the Algerian sample from Tindouf (0.62 ± 0.19) (Table 1). Eleven loci displayed a significant (P<0.05) deviation from HW proportions in the total sample (S3 Table). In all cases except one (CMS18), departure from HW proportions was due to excess of homozygous genotypes. The highest number of significantly (P<0.05) deviating loci (7) was observed for the samples from Tamanrasset (S4 Table).

**Table 1:** Genetic diversity parameters for the total sample (n = 331) arranged by sampling area.

	ALGERIA					EGYPT					
	BEC	STE	TIN	ADR	TAM	ALQ	IKI	BIR	MAR	SID	NEG
**N**_**a**_	5.2 (2.6)	5.8 (3.3)	5.8 (3.3)	5.9 (3.7)	7.7 (4.9)	5.5 (2.4)	6.5 (3.4)	5.3 (2.5)	4.9 (2.3)	5.7 (2.9)	5.6 (2.7)
**A**_**r**_	5.6 (2.4)	5.5 (2.7)	5.3 (2.4)	5.9 (3.0)	5.5 (2.6)	5.5 (2.6)	5.4 (2.5)	5.2 (2.8)	5.1 (2.5)	5.4 (2.6)	5.3 (2.9)
**Gene diversity**	0.63 (0.17)	0.64 (0.19)	0.62 (0.19)	0.64 (0.20)	0.64 (0.19)	0.64 (0.18)	0.64 (0.16)	0.66 (0.15)	0.64 (0.19)	0.63 (0.19)	0.65 (0.15)

N_a_, Number of alleles; A_r_, Allelic richness, estimated on a rarefied sample of 30 “genes” (n = 15 individuals). BEC, Bechar; STE, Steppe; TIN, Tindouf; ADR, Adrar; TAM, Tamanrasset; ALQ, Al Qalaj; IKI, Iking Maryut; BIR, Birqash; MAR, Marsa Matruh; SID, Sidi Barrani; NEG, Negeila.

Values averaged over loci are provided for the considered parameters.

In parentheses, standard deviation values are provided.

Out of 171 possible pair-wise comparisons, only fifteen (8.8%) displayed significant (P<0.01) gametic unbalance in the total sample (data not shown).

### Genetic variability by population

Genetic variability was also investigated in a subset of 185 Algerian and 70 Egyptian samples, for which population information was available (see “Materials and methods” section). As already presented for the total sample and for the dataset arranged in five Algerian and six Egyptian regions, the locus VOLP32 was the most informative (highest *F*_ST_ value, 0.09) (S3 Table), and the loci CVRL8 and CVRL1D displayed the lowest (2) and the highest (18–22) number of alleles also within the six populations considered in this study (S5 Table). No remarkable difference was observed between populations in allelic richness values, that ranged from 5.0 (Azawad) to 5.4 (Targui and Maghraby). Private allelic richness ranged from 0.21 (Rguibi) to 0.55 (Maghraby) (data not shown). The highest gene diversity was observed in the Sudani sample from Egypt (0.66 ±0.15), the lowest being observed in the Rguibi sample from Algeria (0.63±0.19) (S5 Table). The highest number of significantly (P<0.05) deviating loci (7) was observed for the Targui sample (S5 Table). Locus by locus *F*_ST_ values for the dataset arranged into different populations displayed a 0.94 correlation coefficient with locus by locus F_ST_ values for the dataset arranged into different geographic regions (data not shown).

### Genetic relationship among regions

**Fig 2:**
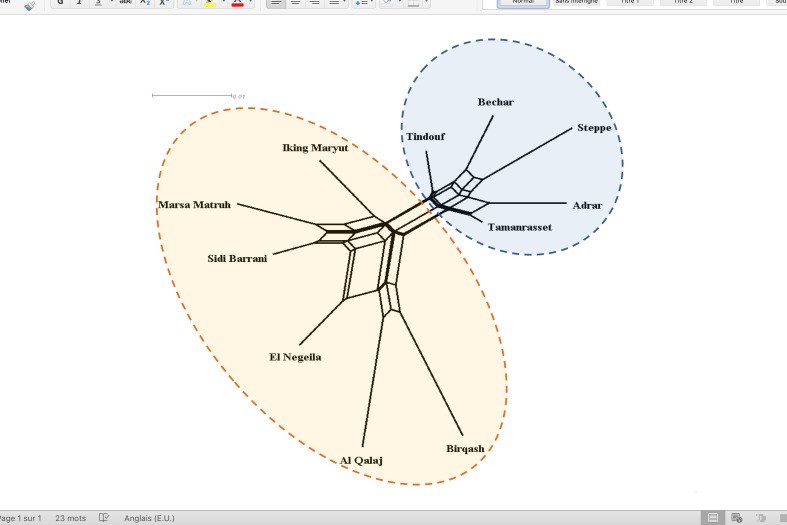
Neighbor-net network constructed using the distance of Reynolds et al. (1983) considering the whole dataset arranged into five Algerian (light blue area) and six Egyptian (light yellow area) geographical regions.

**Fig 3:**
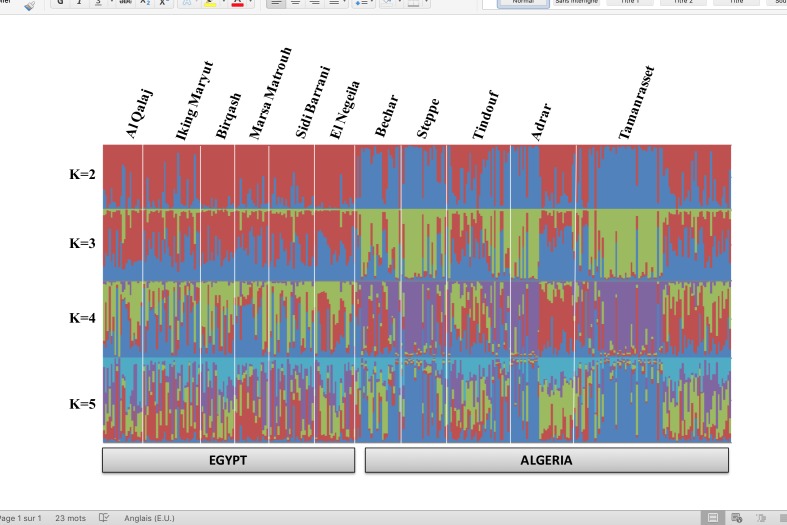
Plot obtained using STRUCTURE’s coefficients of individual membership to clusters (K) assumed to be present in the Algerian and Egyptian samples.

We first investigated genetic relationship among the considered eleven Algerian and Egyptian geographical regions by pair-wise *F*_ST_ (S6 Table). Out of 55 pair-wise comparisons, only 36 (65%) were significant with P<0.01 and differentiation values were generally low. The highest values were observed for the pair-wise comparisons involving samples from Al Qalaj (Egypt), all of them belonging to the Falahy population. Generally low values (0.002–0.012) were observed for pair-wise comparisons involving samples from different Algerian geographic regions, and only two of them (Steppe *vs*. Adrar and Steppe *vs*. Tamanrasset) were statistically significant. The network constructed using the distance of Reynolds *et al*. (1983) grossly mirrored the above results, with Algerian samples displaying a less expanded topology compared to the Egyptian ones, and a clear, though weak, differentiation between samples from the two countries being evident (Fig 2). We then investigated genetic structure using the unsupervised Bayesian clustering approach implemented in the software STRUCTURE. Based on the Evanno’s DeltaK statistics, the most probable number of clusters in our sample was K = 2 (S1 Fig). However, no clearly interpretable sub-structuring was observed at K = 2, nor at higher K values. Indeed, with the exception of the weak differentiation between Algerian and Egyptian samples, the other visible substructures could not be correlated to any geographic sampling area, population, or herd (Fig 3).

### Genetic relationship among populations

In order to test whether the faint differentiation observed between samples from the two countries could be ascribed to differences among camel populations, we estimated pair-wise *F*_ST_ values among the three Egyptian (Falahy, Maghraby, Sudany) and the three Algerian (Azawad, Rguibi, Targui) populations. Although being very low, almost all the pair-wise *F*_ST_ values were significant (P<0.01). Again, differentiation between Egyptian population pairs was higher than Algerian population pairs (S7 Table). Similar results were also evident from the networks constructed using the distance of Nei (1983) and the distance of Reynolds et al. (1983) on the dataset arranged into the three Egyptian and the three Algerian considered populations (S2 and S3 Figs, respectively). The latter also displayed an intermediate position of Maghraby between Algerian and the other two Egyptian populations. The observed network topology (differentiation between Algerian and Egyptian samples, and intermediate position of the Maghraby population between Algerian and the other two Egyptian populations) was strongly supported by NJ tree bootstrap values (S4 and S5 Figs). Both NJ trees also highlighted the pairs Falahi-Sudani and Targui-Azawad. Results from COANCESTRY indicated that sampling was not biased towards highly related individuals. Mean relatedness (R) over the total sample was 0.058 ± 0.077. Within single populations, the majority (96% to 97%) of the pair-wise comparisons among individuals gave TrioML values lower than 0.25 (Unrelated animals), only a limited proportion of pair-wise comparisons (3% to 4%) gave TrioML values > 0.25 and≤0.5 (Half-siblings), and no pair-wise comparison produced TrioML values higher than 0.5 (Full-siblings) (S8 Table). Even more so, when estimating relatedness among individuals from different population pairs, the majority (96% to 98%) of the pair-wise comparisons gave TrioML values lower than 0.25 (Unrelated animals) (S8 Table).

## Discussion

Inventory and characterization of animal genetic resources and monitoring populations for variability are widely recognized as fundamental steps for any breed conservation and improvement programme [44]. Unfortunately, advancements in this field are still scarce for the dromedary (*Camelus dromedarius*) species for which no long-established herd book breed exists and most of the existing populations have not been described yet or they have been only described at the phenotypic level. Compared to major livestock species, a reduced number of studies have been carried out on the genetic characterization of dromedary populations, mainly using microsatellite markers, and generally not trespassing single country sampling areas [45–59]. Very recently, the comprehensive study by Almathen et al. (2016) investigated dynamics of dromedary domestication and cross-continental dispersal combining ancient DNA sequences of wild and early-domesticated dromedary samples from arid regions with nuclear microsatellite and mitochondrial genotype information from 1,083 extant animals collected across the whole species’ geographic range. The study included samples from Algeria (30) and Egypt (30). However, the focus of the work being on cross-continental dispersal, the analyses were carried out on data aggregated into five defined geographical regions: Eastern Africa, Western and Northern Africa, North Arabian Peninsula South Arabian Peninsula, and Southern Asia including Australia. In this study, a survey of the genetic variability and relationships among six dromedary populations from thirteen geographic regions in Algeria and Egypt was carried out on a dataset of 331 animals by using 19 polymorphic microsatellite markers. A generally high level of polymorphism (number of alleles, gene diversity) was observed within the six considered populations, comparable with previous results from five Moroccan populations [49] and three Tunisian populations [50].

### Genetic structure of camels in Algeria largely reflects past geographic distribution of nomadic pastoralist tribes

Consistent with a phylogeographic pattern, a slightly closer relationship of the Steppe region with Bechar and Tindouf (Moroccan border) than with Adrar and Tamanrasset (Mali and Niger border, respectively) was observed for the Algerian samples. This result is also in agreement with distribution of Rguibi dromedaries in the western part of Algeria, close to Morocco. Indeed, Rguibi dromedaries were originally reared by the Reguibat tribe, whose traditional vast nomadic range included, among other, also Morocco, and western Algeria [60]. On the contrary, both Azawad and Targui dromedaries were originally reared by nomadic Touareg people that occupied a vast area including eastern Mali, western Niger, and south-eastern Algeria. Azawad dromedaries, that derive their name from the Azawad territory in northern Mali, are mainly present in Algeria in the wilaya of Adrar (whose southern territory borders Mali). Targui dromedaries, that derive their name from the Touareg people (in Arabic, Targui is the singular form of Touareg), in Algeria are mainly present in the Tamanrasset wilaya (whose southern territory borders Niger).

### Egyptian camel populations: at the intersection between north-west and eastern Africa

In Egypt, four main camel populations are generally recognized, Sudani, Maghrabi, Falahi (or Fellahi, or Baladi) and Mowalled [28]. The latter is a cross between the Maghrabi and the Falahi, and was hence not considered in our study. Among the three considered Egyptian populations, the Maghrabi resulted to be the most differentiated. These results are likely due to its geographical origin from coastal North-West Africa (Maghreb countries), while both Sudani and Falahi would have Eastern Africa origins. The Sudani is indeed imported from Sudan. The Falahi is bred in Upper Egypt but mostly used in the Nile delta region. A study carried out by [48] using few microsatellite markers highlighted a certain level of genetic relationship between Falahi and Somali dromedaries. As predictable based on geography, an intermediate position of Maghraby dromedaries, between the three Algerian and the other two Egyptian population clusters was observed in our study.

### Weak genetic structure at the cross-border level in northern African dromedary camels

The faint sub-structuring observed using the unsupervised Bayesian clustering algorithm when analyzing the whole dataset is seemingly the result of a poor genetic differentiation, also suggested by the low observed *F*_ST_ values. In addition, while samples were *a priori* assigned to a specific population/geographic isolate when performing tree/network analysis, this was not the case when the Bayesian clustering approach was adopted (animals were allocated to assumed clusters by the algorithm). This could explain why a certain degree of differentiation was observable from trees and neighbor-network plots and not from the STRUCTURE plot. Indeed, when neighbor-network plots were obtained using inter-individual distance matrices (data not shown), instead of inter-population distance matrices, the obtained topologies did not suggest any interpretable scenario.

In summary, our results point to a weak genetic differentiation of dromedary populations and geographic isolates in two non-contiguous North African countries (Algeria and Egypt). These results are in agreement with those recently presented in the study by Almathen et al. (2016), where little population structure is observed using microsatellite loci in modern dromedaries, as a consequence of historical use as a cross-continental beast of burden along trans-Saharan caravan routes, coupled to traditional extensive/nomadic herding practices. A weak genetic differentiation has been observed also in the preliminary study by Ciani et al[61], performed using genome-wide SNP markers genotyped by double digest Restriction Associated DNA sequencing (ddRADseq), on African and Asiatic dromedaries. Together with the recent evidence for a low SNP density in dromedary genomes compared to genomes of domestic Bactrian, and even compared to genomes of the very small residual population of wild *Camelus ferus* animals [62], likely due to successive climate-driven demographic bottlenecks in the wild progenitor of *Camelus dromedarius*, this lack of genetic structure deserves close attention in view of the ongoing intensification process in the camel farming system [63] and the growing request for application of modern genetic improvement (bio)technologies. Notably, schemes of genomic selection across multiple populations are likely to represent the future choice for this species, in order to allow larger reference populations to be available (thus accounting for possibly lower linkage disequilibrium levels in dromedaries compared to other livestock species), and to take advantage from implementation of cross-border cost-sharing strategies.

## Supporting Information

S1 Table Sample information. (DOCX)

S2 Table Microsatellite information. (DOC)

S3 Table Locus-by-locus genetic diversity parameters for the total sample (N = 331). (DOCX)

S4 Table Locus-by-locus genetic diversity parameters for the Algerian sample arranged into five geographical regions (A) and for the Egyptian sample arranged into six geographical regions (B).
(DOCX)

S5 Table Locus-by-locus genetic diversity parameters for the Algerian (A) and the Egyptian (B) samples arranged into six different populations.
(DOCX)

S6 Table Pair-wise F_ST_ values among the six Egyptian (Al Qalaj, Iking Maryut, Birqash, Marsa Matruh, Sidi Barrany, Negeila) and the five Algerian (Bechar, Steppe, Tindouf, Adrar, Tamanrasset) considered geographical regions. (DOCX)

S7 Table Pair-wise F_ST_ values among the three Egyptian (Falahy, Maghraby, Sudany) and the three Algerian (Azawad, Rguibi, Targui) populations. (DOCX)

S8 Table Proportions of pair-wise comparisons among individuals falling within the considered classes of relatedness (R). (DOCX)

S1 Fig Plot of the DeltaK statistics (Evanno *et al*., 2005) for the total sample. (DOCX)

S2 Fig Neighbor-net network constructed using the distance of Nei (1983) considering the whole dataset arranged into three Algerian (light blue area) and three Egyptian (light yellow area) populations. (DOCX)

S3 Fig Neighbor-net network constructed using the distance of Reynolds *et al*. (1983) considering the whole dataset arranged into three Algerian (light blue area) and three Egyptian (light yellow area) populations. (DOCX)

S4 Fig Neighbor-joining tree constructed using the distance of Nei (1983) considering the whole dataset arranged into three Algerian and three Egyptian populations. (DOCX)

S5 Fig Neighbor-joining tree constructed using the distance of Reynolds *et al*. (1983) considering the whole dataset arranged into three Algerian and three Egyptian populations. (DOCX)

S1 Dataset Genotypic data for the total population sample typed at 19 microsatellite loci. (XLSX)

## References

[1] Albright WF (1951) The Archaeology of Palestine: Penguin Books.

[2] Mikesell MW (1955) Notes on the dispersal of the dromedary Southwestern Journal of Anthropology: 231–245.

[3] Pritchard JB (1969) The ancient Near East: Supplementary texts and pictures relating to the Old Testament: Princeton University Press.

[4] Rothenberg B (1972) Timna; valley of the Biblical copper mines: Thames and Hudson.

[5] Compagnoni B, Tosi M (1978) The camel: Its distribution and state of domestication in the Middle East during the third millennium BC in light of finds from Shahr-i Sokhta.

[6] Lernau H (1978) Faunal remains,. Early Arad: 83–113.

[7] Hoch E (1979) Reflections on prehistoric life at Umm an-Nar (Trucial Oman), based on faunal remains from the third millennium B.C. South Asian Archaeology. the Fourth International Conference of the association of South Asian archaeologists in Western Europe. the Istututo Universitario Orientale, Naples. pp. 589–638.

[8] Hakker-Orion D (1984) The role of the camel in Israel's early history. Animals and Archaeology 3: 207–212.

[9] Wapnish P (1984) The dromedary and Bactrian camel in Levantine historical settings: the evidence from Tell Jemmeh: BAR.

[10] Ripinsky M (1985) The camel in dynastic Egypt. The Journal of Egyptian Archaeology 71: 134–141.

[11] Zarins J (1989) Pastoralism in southwest Asia: the second Millennium BC. The Walking Larder: Patterns of Domestication, Pastoralism, and Predation London: 127–155.

[12] Macdonald MC (1995) North Arabia in the First Millennium BCE. Civilizations of the Ancient Near East 2: 1355–1369.

[13] Hoyland RG (2001) Arabia and the Arabs: From the Bronze Age to the coming of Islam: Psychology Press.

[14] Von den Driesch A, Obermaier H (2007) The Hunt for Wild Dromedaries During the 3rd and 2nd Millennia BC on the United Arab Emirates Coast. Camel Bone Finds from the Excavations at Al Sufouh 2, Dubai, UAE. entry in: Grupe G/Peters J (eds): Skeletal Series and their Socio-economic Context Documenta Archaeobiologiae 5: 133–167.

[15] Uerpmann H-P, Uerpmann M (2002) The appearance of the domestic camel in south-east Arabia. Journal of Oman Studies 12: 235–260.

[16] Uerpmann UHPa. Animals, Labour and Beasts of Burden in Southeast Arabian Pre—Protohistory In: Potts D. T. & Hellyer P. Fifty Years of Emirates Archaeology PotSICotAotUAE, editor; 2012 Motivate Publishing pp. 78–85

[17] Heide M (2010) The domestication of the camel: biological, archaeological and inscriptional evidence from Mesopotamia, Egypt, Israel and Arabia, and traditional evidence from the Hebrew Bible. Ugarit-Forschungen 42: 331–382.

[18] Grigson C (2012) Camels, copper and donkeys in the early iron age of the southern levant: timna revisited. Levant 44: 82–100.

[19] Sapir-Hen L, Ben-Yosef E (2013) The introduction of domestic camels to the southern Levant: evidence from the Aravah Valley. Tel Aviv 40: 277–285.

[20] Jennings RP, Shipton C, Al-Omari A, Alsharekh AM, Crassard R, et al (2013) Rock art landscapes beside the Jubbah palaeolake, Saudi Arabia. Antiquity 87: 666–683.

[21] Epstein H, Mason IL (1971) Origin of the domestic animals of Africa.

[22] Bulliet (1975) The camel and the wheel Cambridge. MA: Harvard.

[23] Midant-Reynes B, Braunstein-Silvestre F (1977) Le chameau en Egypte. Orientalia Roma 46: 337–362.

[24] Grossi D (2011) Presenze di cammelli nell'Antichità in Italia e in Europa. 91–106 p.

[25] Faye B (2009) L’élevage des grands camélidés: vers un changement de paradigme. Rencontres autour des Recherches sur les Ruminants 16: 345–348.

[26] Faye B, Jaouad M, Bhrawi K, Senoussi A, Bengoumi M (2014) Elevage camelin en Afrique du Nord: état des lieux et perspectives. Revue d’élevage et de médecine vétérinaire des pays tropicaux 67: 213–221.

[27] FAOstat (2013).

[28] Mukasa-Mugerwa E (1981) The camel (Camelus dromedarius): A bibliographical review: ILRI (aka ILCA and ILRAD).

[29] Aissa Ben (1989) Le dromadaire en Algérie. Options Méditerranéennes 2: 19–28.

[30] Youcef amine Cherifi, Semir GSB, Nasreddine M, Aoul NT, Saïdi-Mehtar N (2013) Study of Camelina Biodiversity in Southwestern of Algeria. Journal of Life Sciences 7: 416.

[31] Peter C, Bruford M, Perez T, Dalamitra S, Hewitt G, et al (2007) Genetic diversity and subdivision of 57 European and Middle‐Eastern sheep breeds. Animal genetics 38: 37–44. 10.1111/j.1365-2052.2007.01561.x 17257186

[32] FAO (2011) Molecular genetic characterization of animal genetic resources. FAO Animal Production and Health Guidelines. pp. 85.

[33] Almathen F, Charruau P, Mohandesan E, Mwacharo JM, Orozco-terWengel P, et al (2016) Ancient and modern DNA reveal dynamics of domestication and cross-continental dispersal of the dromedary. Proceedings of the National Academy of Sciences: 201519508. 10.1073/pnas.1519508113 PMC4914195 27162355

[34] Excoffier L, Lischer HEL (2010) Arlequin suite ver 3.5: a new series of programs to perform population genetics analyses under Linux and Windows. Molecular Ecology Resources 10: 564–567. 10.1111/j.1755-0998.2010.02847.x 21565059

[35] Kalinowski ST (2005) Hp‐rare 1.0: a computer program for performing rarefaction on measures of allelic richness. Molecular Ecology Notes 5: 187–189.

[36] Pritchard JK, Stephens M, Donnelly P (2000) Inference of population structure using multilocus genotype data. Genetics 155: 945–959. 10835412 10.1093/genetics/155.2.945 PMC1461096

[37] Earl DA, vonHoldt BM (2011) STRUCTURE HARVESTER: a website and program for visualizing STRUCTURE output and implementing the Evanno method. Conservation Genetics Resources 4: 359–361.

[38] Evanno G, Regnaut S, Goudet J (2005) Detecting the number of clusters of individuals using the software STRUCTURE: a simulation study. Molecular ecology 14: 2611–2620. 10.1111/j.1365-294X.2005.02553.x 15969739

[39] Reynolds J, Weir BS, Cockerham CC (1983) Estimation of the coancestry coefficient: basis for a short-term genetic distance. Genetics 105: 767–779. 17246175 10.1093/genetics/105.3.767 PMC1202185

[40] Liu K, Muse SV (2005) PowerMarker: an integrated analysis environment for genetic marker analysis. Bioinformatics 21: 2128–2129. 10.1093/bioinformatics/bti282 15705655

[41] Bryant D, Moulton V (2004) Neighbor-net: an agglomerative method for the construction of phylogenetic networks. Molecular biology and evolution 21: 255–265. 10.1093/molbev/msh018 14660700

[42] Huson DH, Bryant D (2006) Application of phylogenetic networks in evolutionary studies. Molecular biology and evolution 23: 254–267. 10.1093/molbev/msj030 16221896

[43] Wang J (2011) COANCESTRY: a program for simulating, estimating and analysing relatedness and inbreeding coefficients. Molecular ecology resources 11: 141–145. 10.1111/j.1755-0998.2010.02885.x 21429111

[44] FAO (2007) Global Plan of Action for Animal Genetic Resources and the Intertaken Declaration: FAO.

[45] Almathen F, Mwaracharo J, Hanotte O (2012) Genetic diversity and relationships of indigenous Saudi Arabia camel Camelus dromedarius populations. EH Johnson et al(Eds): 40–41.

[46] Mahmoud A, Alshaikh M, Aljumaah R, Mohammed O (2012) Genetic variability of camel (Camelus dromedarius) populations in Saudi Arabia based on microsatellites analysis. African Journal of Biotechnology 11: 11173–11180.

[47] Mahmoud A, AlShaikh M, Aljummah R, Mohammed O (2013) Genetic characterization of Majaheem camel population in Saudi Arabia based on microsatellite markers, Res. J Biotechnol 8: 26–30.

[48] Mahrous KF, Ramadan HA, Abdel-Aziem SH, Abd-El Mordy M, Hemdan DM (2011) Genetic variations between camel breeds using microsatellite markers and RAPD techniques. J Appl Biosci 39: 2626–2634.

[49] Piro M, Bouazzati O, Bengoumi M, El Allali K, Achaaban M, et al (2011) Genetic characterisation of moroccan camel populations using microsatellites markers. Journal of Camel Practice and Research 18: 167–172.

[50] Ould Ahmed M, Salem FB, Bedhiaf S, Rekik B, Djemali M (2010) Genetic diversity in Tunisian dromedary (Camelus dromedarius) populations using microsatellite markers. Livestock Science 132: 182–185.

[51] Spencer P, Woolnough A (2010) Assessment and genetic characterisation of Australian camels using microsatellite polymorphisms. Livestock Science 129: 241–245.

[52] Al-Swailem AM, Shehata MM, Al-Busadah KA, Fallatah MH, Askari E (2009) Evaluation of the genetic variability of microsatellite markers in Saudi Arabian camels. International journal of food, agriculture and environment 7: 636–639.

[53] Mehta S, Goyal A, Sahani M (2007) Microsatellite markers for genetic characterisation of Kachchhi camel. Indian Journal of Biotechnology 6: 336–339.

[54] Vijh R, Tantia M, Mishra B, Bharani Kumar S (2007) Genetic diversity and differentiation of dromedarian camel of India. Animal biotechnology 18: 81–90. 10.1080/10495390600648741 17453647

[55] Nolte M, Kotze A, Van der Bank F, Grobler J (2005) Microsatellite markers reveal low genetic differentiation among southern African Camelus dromedarius populations. South African Journal of Animal Science 35: 152–161.

[56] Schulz U, Minguez Y, Checa M, Garcia-Atance P, Dunner S, et al (2005) The Majorero camel (Camelus dromedarius) breed. Animal Genetic Resources Information 36: 61–71.

[57] Gautam L, Mehta S, Gahlot R, Gautam K (2004) Genetic characterisation of Jaisalmeri camel using microsatellite markers. Indian Journal of Biotechnology 3: 457–459.

[58] Mburu D, Ochieng J, Kuria S, Jianlin H, Kaufmann B, et al (2003) Genetic diversity and relationships of indigenous Kenyan camel (Camelus dromedarius) populations: implications for their classification. Animal Genetics 34: 26–32. 12580783 10.1046/j.1365-2052.2003.00937.x

[59] Evdotchenko D, Han Y, Bartenschlager H, Preuss S, Geldermann H (2003) New polymorphic microsatellite loci for different camel species. Molecular Ecology Notes 3: 431–434.

[60] Olson JS (1996) The peoples of Africa: an ethnohistorical dictionary: Greenwood Publishing Group.

[61] Ciani E BP. First insight on the genetic structure of Camelus dromedarius populations through genome-wide SNP markersICAR Satellite conference on Camelid Reproduction; 2016 1–3 july; France (Tours).

[62] Fitak RR, Mohandesan E, Corander J, Burger PA (2016) The de novo genome assembly and annotation of a female domestic dromedary of North African origin. Molecular ecology resources 16: 314–324. 10.1111/1755-0998.12443 26178449 PMC4973839

[63] Faye B (2016) The camel, new challenges for a sustainable development. Tropical Animal Health and Production: 1–4. 10.1007/s11250-016-0995-8 PMC7088623 26922737

