# Supplementary material for: Weak Genetic Structure in Northern African Dromedary Camels Reflects Their Unique Evolutionary History
Source: PLoS One. 2017 Jan 19;12(1):e0168672. doi: 10.1371/journal.pone.0168672 (PMC5245891; doi:10.1371/journal.pone.0168672)
Supplement: S1 Table — (DOCX) [file pone.0168672.s001.docx]

| **ID** | **Country** | **Sampling site** | **Population** |
| --- | --- | --- | --- |
| 1 | Egypt | Al Qalaj | Falahy |
| 2 | Egypt | Al Qalaj | Falahy |
| 3 | Egypt | Al Qalaj | Falahy |
| 4 | Egypt | Al Qalaj | Falahy |
| 5 | Egypt | Al Qalaj | Falahy |
| 6 | Egypt | Al Qalaj | Falahy |
| 7 | Egypt | Al Qalaj | Falahy |
| 8 | Egypt | Al Qalaj | Falahy |
| 9 | Egypt | Al Qalaj | Falahy |
| 10 | Egypt | Al Qalaj | Falahy |
| 11 | Egypt | Al Qalaj | Falahy |
| 12 | Egypt | Al Qalaj | Falahy |
| 13 | Egypt | Al Qalaj | Falahy |
| 14 | Egypt | Al Qalaj | Falahy |
| 15 | Egypt | Al Qalaj | Falahy |
| 16 | Egypt | Al Qalaj | Falahy |
| 17 | Egypt | Al Qalaj | Falahy |
| 18 | Egypt | Al Qalaj | Falahy |
| 19 | Egypt | Al Qalaj | Falahy |
| 20 | Egypt | Al Qalaj | Falahy |
| 21 | Egypt | Al Qalaj | Falahy |
| 22 | Egypt | Al Qalaj | Falahy |
| 23 | Egypt | Al Qalaj | Falahy |
| 24 | Egypt | Iking Maryut | Maghraby |
| 25 | Egypt | Iking Maryut | Maghraby |
| 26 | Egypt | Iking Maryut | Maghraby |
| 27 | Egypt | Iking Maryut | Maghraby |
| 28 | Egypt | Iking Maryut | Maghraby |
| 29 | Egypt | Iking Maryut | Maghraby |
| 30 | Egypt | Iking Maryut | Maghraby |
| 31 | Egypt | Iking Maryut | Maghraby |
| 32 | Egypt | Iking Maryut | Maghraby |
| 33 | Egypt | Iking Maryut | Maghraby |
| 34 | Egypt | Iking Maryut | Maghraby |
| 35 | Egypt | Iking Maryut | Maghraby |
| 36 | Egypt | Iking Maryut | Maghraby |
| 37 | Egypt | Iking Maryut | Maghraby |
| 38 | Egypt | Iking Maryut | Maghraby |
| 39 | Egypt | Iking Maryut | Maghraby |
| 40 | Egypt | Iking Maryut | Maghraby |
| 41 | Egypt | Iking Maryut | Maghraby |
| 42 | Egypt | Iking Maryut | Maghraby |
| 43 | Egypt | Iking Maryut | Maghraby |
| 44 | Egypt | Iking Maryut | Maghraby |
| 45 | Egypt | Iking Maryut | Maghraby |
| 46 | Egypt | Iking Maryut | Maghraby |
| 47 | Egypt | Iking Maryut | Maghraby |
| 48 | Egypt | Iking Maryut | Maghraby |
| 49 | Egypt | Iking Maryut | Maghraby |
| 50 | Egypt | Iking Maryut | Maghraby |
| 51 | Egypt | Iking Maryut | Maghraby |
| 52 | Egypt | Iking Maryut | Maghraby |
| 53 | Egypt | Iking Maryut | Maghraby |
| 54 | Egypt | Birqash | Sudany |
| 55 | Egypt | Birqash | Sudany |
| 56 | Egypt | Birqash | Sudany |
| 57 | Egypt | Birqash | Sudany |
| 58 | Egypt | Birqash | Sudany |
| 59 | Egypt | Birqash | Sudany |
| 60 | Egypt | Birqash | Sudany |
| 61 | Egypt | Birqash | Sudany |
| 62 | Egypt | Birqash | Sudany |
| 63 | Egypt | Birqash | Sudany |
| 64 | Egypt | Birqash | Sudany |
| 65 | Egypt | Birqash | Sudany |
| 66 | Egypt | Birqash | Sudany |
| 67 | Egypt | Birqash | Sudany |
| 68 | Egypt | Birqash | Sudany |
| 69 | Egypt | Birqash | Sudany |
| 70 | Egypt | Birqash | Sudany |
| 71 | Egypt | Mersa Matrouh | ND |
| 72 | Egypt | Mersa Matrouh | ND |
| 73 | Egypt | Mersa Matrouh | ND |
| 74 | Egypt | Mersa Matrouh | ND |
| 75 | Egypt | Mersa Matrouh | ND |
| 76 | Egypt | Mersa Matrouh | ND |
| 77 | Egypt | Mersa Matrouh | ND |
| 78 | Egypt | Mersa Matrouh | ND |
| 79 | Egypt | Mersa Matrouh | ND |
| 80 | Egypt | Mersa Matrouh | ND |
| 81 | Egypt | Mersa Matrouh | ND |
| 82 | Egypt | Mersa Matrouh | ND |
| 83 | Egypt | Mersa Matrouh | ND |
| 84 | Egypt | Mersa Matrouh | ND |
| 85 | Egypt | Mersa Matrouh | ND |
| 86 | Egypt | Mersa Matrouh | ND |
| 87 | Egypt | Sedi-Barrany | ND |
| 88 | Egypt | Sedi-Barrany | ND |
| 89 | Egypt | Sedi-Barrany | ND |
| 90 | Egypt | Sedi-Barrany | ND |
| 91 | Egypt | Sedi-Barrany | ND |
| 92 | Egypt | Sedi-Barrany | ND |
| 93 | Egypt | Sedi-Barrany | ND |
| 94 | Egypt | Sedi-Barrany | ND |
| 95 | Egypt | Sedi-Barrany | ND |
| 96 | Egypt | Sedi-Barrany | ND |
| 97 | Egypt | Sedi-Barrany | ND |
| 98 | Egypt | Sedi-Barrany | ND |
| 99 | Egypt | Sedi-Barrany | ND |
| 100 | Egypt | Sedi-Barrany | ND |
| 101 | Egypt | Sedi-Barrany | ND |
| 102 | Egypt | Sedi-Barrany | ND |
| 103 | Egypt | Sedi-Barrany | ND |
| 104 | Egypt | Sedi-Barrany | ND |
| 105 | Egypt | Sedi-Barrany | ND |
| 106 | Egypt | Sedi-Barrany | ND |
| 107 | Egypt | Sedi-Barrany | ND |
| 108 | Egypt | Sedi-Barrany | ND |
| 109 | Egypt | Sedi-Barrany | ND |
| 110 | Egypt | Sedi-Barrany | ND |
| 111 | Egypt | Negeila | ND |
| 112 | Egypt | Negeila | ND |
| 113 | Egypt | Negeila | ND |
| 114 | Egypt | Negeila | ND |
| 115 | Egypt | Negeila | ND |
| 116 | Egypt | Negeila | ND |
| 117 | Egypt | Negeila | ND |
| 118 | Egypt | Negeila | ND |
| 119 | Egypt | Negeila | ND |
| 120 | Egypt | Negeila | ND |
| 121 | Egypt | Negeila | ND |
| 122 | Egypt | Negeila | ND |
| 123 | Egypt | Negeila | ND |
| 124 | Egypt | Negeila | ND |
| 125 | Egypt | Negeila | ND |
| 126 | Egypt | Negeila | ND |
| 127 | Egypt | Negeila | ND |
| 128 | Egypt | Negeila | ND |
| 129 | Egypt | Negeila | ND |
| 130 | Egypt | Negeila | ND |
| 131 | Egypt | Negeila | ND |
| 132 | Egypt | Negeila | ND |
| 133 | Egypt | Negeila | ND |
| 134 | Algeria | Bechar | Rguibi |
| 135 | Algeria | Bechar | Rguibi |
| 136 | Algeria | Bechar | Rguibi |
| 137 | Algeria | Bechar | Rguibi |
| 138 | Algeria | Bechar | Rguibi |
| 139 | Algeria | Bechar | Rguibi |
| 140 | Algeria | Bechar | Rguibi |
| 141 | Algeria | Bechar | Rguibi |
| 142 | Algeria | Bechar | Rguibi |
| 143 | Algeria | Bechar | Rguibi |
| 144 | Algeria | Bechar | Azawad |
| 145 | Algeria | Bechar | Azawad |
| 146 | Algeria | Bechar | Azawad |
| 147 | Algeria | Bechar | Azawad |
| 148 | Algeria | Bechar | Azawad |
| 149 | Algeria | Bechar | Rguibi |
| 150 | Algeria | Bechar | Rguibi |
| 151 | Algeria | Bechar | Azawad |
| 152 | Algeria | Bechar | Azawad |
| 153 | Algeria | Bechar | ND |
| 154 | Algeria | Bechar | Rguibi |
| 155 | Algeria | Bechar | Rguibi |
| 156 | Algeria | Steppe | ND |
| 157 | Algeria | Steppe | ND |
| 158 | Algeria | Steppe | ND |
| 159 | Algeria | Steppe | Rguibi |
| 160 | Algeria | Steppe | ND |
| 161 | Algeria | Steppe | Rguibi |
| 162 | Algeria | Steppe | Rguibi |
| 163 | Algeria | Steppe | Rguibi |
| 164 | Algeria | Steppe | Azawad |
| 165 | Algeria | Steppe | Azawad |
| 166 | Algeria | Steppe | Rguibi |
| 167 | Algeria | Steppe | ND |
| 168 | Algeria | Steppe | Rguibi |
| 169 | Algeria | Steppe | Rguibi |
| 170 | Algeria | Steppe | ND |
| 171 | Algeria | Steppe | ND |
| 172 | Algeria | Steppe | Azawad |
| 173 | Algeria | Steppe | ND |
| 174 | Algeria | Steppe | ND |
| 175 | Algeria | Steppe | ND |
| 176 | Algeria | Steppe | Rguibi |
| 177 | Algeria | Steppe | ND |
| 178 | Algeria | Steppe | Rguibi |
| 179 | Algeria | Steppe | ND |
| 180 | Algeria | Tindouf | Rguibi |
| 181 | Algeria | Tindouf | Rguibi |
| 182 | Algeria | Tindouf | Rguibi |
| 183 | Algeria | Tindouf | Rguibi |
| 184 | Algeria | Tindouf | Rguibi |
| 185 | Algeria | Tindouf | Rguibi |
| 186 | Algeria | Tindouf | Rguibi |
| 187 | Algeria | Tindouf | Rguibi |
| 188 | Algeria | Tindouf | Rguibi |
| 189 | Algeria | Tindouf | Rguibi |
| 190 | Algeria | Tindouf | Rguibi |
| 191 | Algeria | Tindouf | Rguibi |
| 192 | Algeria | Tindouf | Rguibi |
| 193 | Algeria | Tindouf | Rguibi |
| 194 | Algeria | Tindouf | Rguibi |
| 195 | Algeria | Tindouf | Rguibi |
| 196 | Algeria | Tindouf | Azawad |
| 197 | Algeria | Tindouf | Azawad |
| 198 | Algeria | Tindouf | Rguibi |
| 199 | Algeria | Tindouf | Rguibi |
| 200 | Algeria | Tindouf | Rguibi |
| 201 | Algeria | Tindouf | Rguibi |
| 202 | Algeria | Tindouf | Rguibi |
| 203 | Algeria | Tindouf | Rguibi |
| 204 | Algeria | Tindouf | Rguibi |
| 205 | Algeria | Tindouf | Rguibi |
| 206 | Algeria | Tindouf | Rguibi |
| 207 | Algeria | Tindouf | Rguibi |
| 208 | Algeria | Tindouf | Rguibi |
| 209 | Algeria | Tindouf | Rguibi |
| 210 | Algeria | Tindouf | Rguibi |
| 211 | Algeria | Tindouf | Rguibi |
| 212 | Algeria | Tindouf | Rguibi |
| 213 | Algeria | Tindouf | Rguibi |
| 214 | Algeria | ADRAR | Rguibi |
| 215 | Algeria | ADRAR | Azawad |
| 216 | Algeria | ADRAR | Azawad |
| 217 | Algeria | ADRAR | Azawad |
| 218 | Algeria | ADRAR | Azawad |
| 219 | Algeria | ADRAR | Azawad |
| 220 | Algeria | ADRAR | Azawad |
| 221 | Algeria | ADRAR | Azawad |
| 222 | Algeria | ADRAR | Azawad |
| 223 | Algeria | ADRAR | Azawad |
| 224 | Algeria | ADRAR | Azawad |
| 225 | Algeria | ADRAR | Azawad |
| 226 | Algeria | ADRAR | Azawad |
| 227 | Algeria | ADRAR | Azawad |
| 228 | Algeria | ADRAR | Azawad |
| 229 | Algeria | ADRAR | Azawad |
| 230 | Algeria | ADRAR | Azawad |
| 231 | Algeria | ADRAR | Azawad |
| 232 | Algeria | ADRAR | Azawad |
| 233 | Algeria | ADRAR | Azawad |
| 234 | Algeria | ADRAR | Azawad |
| 235 | Algeria | ADRAR | Azawad |
| 236 | Algeria | ADRAR | Azawad |
| 237 | Algeria | ADRAR | Azawad |
| 238 | Algeria | ADRAR | Azawad |
| 239 | Algeria | ADRAR | Azawad |
| 240 | Algeria | ADRAR | Azawad |
| 241 | Algeria | ADRAR | Azawad |
| 242 | Algeria | ADRAR | Azawad |
| 243 | Algeria | ADRAR | Targui |
| 244 | Algeria | ADRAR | Targui |
| 245 | Algeria | TAMANRASSET | Targui |
| 246 | Algeria | TAMANRASSET | Targui |
| 247 | Algeria | TAMANRASSET | Targui |
| 248 | Algeria | TAMANRASSET | Targui |
| 249 | Algeria | TAMANRASSET | Targui |
| 250 | Algeria | TAMANRASSET | Targui |
| 251 | Algeria | TAMANRASSET | Targui |
| 252 | Algeria | TAMANRASSET | Targui |
| 253 | Algeria | TAMANRASSET | Targui |
| 254 | Algeria | TAMANRASSET | Targui |
| 255 | Algeria | TAMANRASSET | Targui |
| 256 | Algeria | TAMANRASSET | Targui |
| 257 | Algeria | TAMANRASSET | Targui |
| 258 | Algeria | TAMANRASSET | Targui |
| 259 | Algeria | TAMANRASSET | Targui |
| 260 | Algeria | TAMANRASSET | Targui |
| 261 | Algeria | TAMANRASSET | Targui |
| 262 | Algeria | TAMANRASSET | Targui |
| 263 | Algeria | TAMANRASSET | Targui |
| 264 | Algeria | TAMANRASSET | Targui |
| 265 | Algeria | TAMANRASSET | Targui |
| 266 | Algeria | TAMANRASSET | Targui |
| 267 | Algeria | TAMANRASSET | Targui |
| 268 | Algeria | TAMANRASSET | Targui |
| 269 | Algeria | TAMANRASSET | Targui |
| 270 | Algeria | TAMANRASSET | Targui |
| 271 | Algeria | TAMANRASSET | Targui |
| 272 | Algeria | TAMANRASSET | Targui |
| 273 | Algeria | TAMANRASSET | Targui |
| 274 | Algeria | TAMANRASSET | Targui |
| 275 | Algeria | TAMANRASSET | Targui |
| 276 | Algeria | TAMANRASSET | Targui |
| 277 | Algeria | TAMANRASSET | Targui |
| 278 | Algeria | TAMANRASSET | Targui |
| 279 | Algeria | TAMANRASSET | Targui |
| 280 | Algeria | TAMANRASSET | Targui |
| 281 | Algeria | TAMANRASSET | Targui |
| 282 | Algeria | TAMANRASSET | Targui |
| 283 | Algeria | TAMANRASSET | Targui |
| 284 | Algeria | TAMANRASSET | Targui |
| 285 | Algeria | TAMANRASSET | Targui |
| 286 | Algeria | TAMANRASSET | Targui |
| 287 | Algeria | TAMANRASSET | Targui |
| 288 | Algeria | TAMANRASSET | Targui |
| 289 | Algeria | TAMANRASSET | Targui |
| 290 | Algeria | TAMANRASSET | Targui |
| 291 | Algeria | TAMANRASSET | Targui |
| 292 | Algeria | TAMANRASSET | Targui |
| 293 | Algeria | TAMANRASSET | Targui |
| 294 | Algeria | TAMANRASSET | Targui |
| 295 | Algeria | TAMANRASSET | Targui |
| 296 | Algeria | TAMANRASSET | Targui |
| 297 | Algeria | TAMANRASSET | Targui |
| 298 | Algeria | TAMANRASSET | Targui |
| 299 | Algeria | TAMANRASSET | Targui |
| 300 | Algeria | TAMANRASSET | Targui |
| 301 | Algeria | TAMANRASSET | Targui |
| 302 | Algeria | TAMANRASSET | Targui |
| 303 | Algeria | TAMANRASSET | Targui |
| 304 | Algeria | TAMANRASSET | Targui |
| 305 | Algeria | TAMANRASSET | Targui |
| 306 | Algeria | TAMANRASSET | Targui |
| 307 | Algeria | TAMANRASSET | Targui |
| 308 | Algeria | TAMANRASSET | Targui |
| 309 | Algeria | TAMANRASSET | Targui |
| 310 | Algeria | TAMANRASSET | Targui |
| 311 | Algeria | TAMANRASSET | Targui |
| 312 | Algeria | TAMANRASSET | Targui |
| 313 | Algeria | TAMANRASSET | Targui |
| 314 | Algeria | TAMANRASSET | Targui |
| 315 | Algeria | TAMANRASSET | Targui |
| 316 | Algeria | TAMANRASSET | Targui |
| 317 | Algeria | TAMANRASSET | Targui |
| 318 | Algeria | TAMANRASSET | Targui |
| 319 | Algeria | TAMANRASSET | Targui |
| 320 | Algeria | TAMANRASSET | Targui |
| 321 | Algeria | TAMANRASSET | Targui |
| 322 | Algeria | TAMANRASSET | Targui |
| 323 | Algeria | TAMANRASSET | Targui |
| 324 | Algeria | TAMANRASSET | Targui |
| 325 | Algeria | TAMANRASSET | Targui |
| 326 | Algeria | TAMANRASSET | Targui |
| 327 | Algeria | TAMANRASSET | Targui |
| 328 | Algeria | TAMANRASSET | Targui |
| 329 | Algeria | TAMANRASSET | Targui |
| 330 | Algeria | TAMANRASSET | Targui |
| 331 | Algeria | TAMANRASSET | Targui |

**Table S1.** Sample information.
