# Supplementary material for: Weak Genetic Structure in Northern African Dromedary Camels Reflects Their Unique Evolutionary History
Source: PLoS One. 2017 Jan 19;12(1):e0168672. doi: 10.1371/journal.pone.0168672 (PMC5245891; doi:10.1371/journal.pone.0168672)
Supplement: S2 Table — (DOC) [file pone.0168672.s002.doc]

| **Locus name** | **GenBank ID** | **Primer sequence** | **Allelic range^1^** | **Dye^2^** | **Multiplex #** | **References** |
| --- | --- | --- | --- | --- | --- | --- |
| CVRL4D | AF217604 | FOR: 5'-[HEX]CCCTACCTCTGGACTTTG-3' | 105-127 (158–178) | G | 1 | Mariasegaram *et al*. (2002) |
|  |  | REV: CCTTTTTGGGTATTTTCAG |  |  |  |  |
| CVRL5D | AF217605 | FOR:5'-[6FAM]CCTTGGACCTCCTTGCTCTG-3’ | 105-130 (148-174) | B | 1 | Mariasegaram *et al* .(2002) |
|  |  | REV:GCCACTGGTCCCTGTCATT |  |  |  |  |
| CMS121 | AF329159 | FOR: 5'-[6FAM]CAAGAGAACTGGTGAGGATTTTC-3' | 147-173 (147–166) | B | 1 | Evdotchenko *et al*. (2003) |
|  |  | REV: AGTTGATAAAAATACAGCTGGAAAG |  |  |  |  |
| CVRL6D | AF217606 | FOR:5'-HEX] TTTTAAAAATTCTGACCAGGAGTCTG-3’ | 155-175 (185-205) | G | 1 | Mariasegaram *et al*. (2002) |
|  |  | REV:CATAATAGCCAAAACATGGAAACAAC |  |  |  |  |
| LCA66 | AF091125 | FOR:5'-HEX]GTGCAGCGTCCAAATAGTCA-3' | 226-246 (212-262) | G | 1 | Penedo *et al*. (1999) |
|  |  | REV:CCAGCATCGTCCAGTATTCA |  |  |  |  |
| CVRL1D | AF217601 | FOR:5'-[HEX]GAAGAGGTTGGGGCACTAC-3' | 279-325 (188-253) | G | 1 | Mariasegaram *et al*. (2002) |
|  |  | REV: CAGGCAGATATCCATTGAA |  |  |  |  |
| YWLL44 | NF | FOR:5'-[6FAM]CTCAACAATGCTAGACCTTGG-3' | 88-125 (86-120) | B | 2 | Lang *et al*. (1996) |
|  |  | REV:GAGAACACAGGCTGGTGAATA |  |  |  |  |
| YWLL59 | NF | FOR:5-[HEX]'TGTGCAGGAGTTAGGTGTA-3' | 103-268 (107-109) | G | 2 | Lang *et al.* (1996) |
|  |  | REV:CCATGTCTCTGAAGCTCTGGA |  |  |  |  |
| CMS50 | AF329149 | FOR: 5'-[6FAM] TTTATAGTCAGAGAGAGTGCTG-3' | 145-190 (129-190) | B | 2 | Evdotchenko *et al.* (2003) |
|  |  | REV:TGTAGGGTTCATTGTAACA |  |  |  |  |
| CVRL8D | AF217608 | FOR: 5'[6FAM]-AATTCCTGTGATTTTATACACA-3' | 191-209 (207–209) | B | 2 | Mariasegaram *et al*. (2002) |
|  |  | REV: CATGTCATGAAAGCTACAGTA |  |  |  |  |
| CMS09 | AF329160 | FOR:5'-[6FAM]TGCTTTAGACGACTTTTACTTTAC-3' | 225-250 (227-256 ) | B | 2 | Evdotchenko *et al*. (2003) |
|  |  | REV: ATTTCACTTTCTTCATACTTGTGAT |  |  |  |  |
| VOLP10 | AF305231 | FOR:5'[HEX] CTTTCTCCTTTCCTCCCTACT-3’ | 236-268 (250–268) | G | 2 | Obreque *et al*. (1998) |
|  |  | REV : CGTCCACTTCCTTCATTTC |  |  |  |  |
| CVRL7D | AF217607 | FOR:5'[6FAM] AATACCCTAGTTGAAGCTCTGTCCT-3’ | 272-314 (272–306) | B | 2 | Mariasegaram *et al*. (2002) |
|  |  | REV:GAGTGCCTTTATAAATATGGGTCTG |  |  |  |  |
| CMS25 | AF380345 | FOR: 5'-[HEX]GATCCTCCTGCGTTCTTATT-3' | 90-110 (93–102) | G | 3 | Evdotchenko *et al*. (2003) |
|  |  | REV: CTAGCCTTTGATTGGAGCAT |  |  |  |  |
| CMS15 | AF329151 | FOR:5'-[6FAM]AAATACTTAAAGGTTCCCAGA-3' | 116-144 (121-144) | B | 3 | Evdotchenko *et al*. (2003) |
|  |  | REV: TTGTAAACTAAAGCCAGAAAG |  |  |  |  |
| CMS17 | AF329147 | FOR:5'[HEX]TATAAAGGATCACTGCCTTC-3' | 149-170 (135-167) | G | 3 | Evdotchenko *et al*. (2003) |
|  |  | REV:AAAATGAACCTCCATAAAGTTAG |  |  |  |  |
| CMS18 | AF329148 | FOR:5-'[6FAM] GAACGACCCTTGAAGACGAA-3' | 157-170 (157–163) | B | 3 | Evdotchenko *et al*. (2003) |
|  |  | REV:AGCAGCTGGTTTTAGGTCCA |  |  |  |  |
| CMS32 | AF329146 | FOR:5'-[HEX]ACGGACAAGAACTGCTCATA-3' | 198-209 (198-209) | G | 3 | Evdotchenko *et al*. (2003) |
|  |  | REV:ACAACCAATAAATCCCCATT |  |  |  |  |
| CMS13 | AF329158 | FOR: 5'-[6FAM] TAGCCTGACTCTATCCATTTCTC-3’ | 236-260 (238–254) | B | 3 | Evdotchenko *et al*. (2003) |
|  |  | REV:ATTATTTGGAATTCAACTGTAAGG |  |  |  |  |
| VOLP32 | AF305234 | FOR:5'[HEX] GTGATCGGAATGGCTTGAAA-3’ | 256-290 (192-262) | G | 3 | Obreque *et al*. (1998) |
|  |  | REV:CAGCGAGCACCTGAAAGAA |  |  |  |  |

**Table S2.** Microsatellite information.

^1^In parentheses, the allelic ranges reported by the cited literature. ^2^Dyes: B, Blue (6-FAM^TM^): G, Green (HEX^TM^).
