# Supplementary material for: Weak Genetic Structure in Northern African Dromedary Camels Reflects Their Unique Evolutionary History
Source: PLoS One. 2017 Jan 19;12(1):e0168672. doi: 10.1371/journal.pone.0168672 (PMC5245891; doi:10.1371/journal.pone.0168672)
Supplement: S3 Table — (DOCX) [file pone.0168672.s003.docx]

| Locus name | N_a_ | H_E_ | H_O_ | *F*_IS_ | *F*_ST_^(1)^ | P^(1)^ | *F*_ST_^(2)^ | P^(2)^ | |
| --- | --- | --- | --- | --- | --- | --- | --- | --- | --- |
| CVRL4D | 9 | 0.665 | 0.632** | 0.199 | 0.01 | 0.12 | 0.01 | 0.18 |  |
| CVRL5D | 13 | 0.684 | 0.625 | 0.086** | 0.02 | 0.01 | 0.02 | 0.00 |  |
| CMS121 | 12 | 0.723 | 0.722** | 0.002 | 0.01 | 0.04 | 0.01 | 0.01 |  |
| CVRL6D | 4 | 0.342 | 0.332 | 0.029 | 0.01 | 0.15 | 0.01 | 0.04 |  |
| LCA66 | 9 | 0.731 | 0.769 | 0.052 | 0.01 | 0.08 | 0.00 | 0.20 |  |
| CVRL1D | 24 | 0.849 | 0.819 | 0.036* | 0.01 | 0.02 | 0.00 | 0.08 |  |
| YWLL44 | 8 | 0.572 | 0.557* | 0.026 | 0.02 | 0.00 | 0.03 | 0.00 |  |
| YWLL59 | 4 | 0.501 | 0.433** | 0.136* | 0.03 | 0.01 | 0.03 | 0.00 |  |
| CMS50 | 14 | 0.837 | 0.830 | 0.007 | 0.01 | 0.02 | 0.01 | 0.01 |  |
| CVRL8 | 2 | 0.380 | 0.363 | 0.043 | 0.00 | 0.62 | 0.00 | 0.57 |  |
| CMS9 | 11 | 0.812 | 0.636** | 0.217** | 0.04 | 0.00 | 0.05 | 0.00 |  |
| VOLP10 | 13 | 0.803 | 0.739** | 0.080** | 0.00 | 0.63 | 0.01 | 0.06 |  |
| CVRL7 | 16 | 0.807 | 0.758* | 0.061** | 0.02 | 0.00 | 0.02 | 0.00 |  |
| CMS25 | 6 | 0.618 | 0.625 | 0.010 | 0.01 | 0.02 | 0.01 | 0.10 |  |
| CMS15 | 11 | 0.782 | 0.751 | 0.039 | 0.01 | 0.02 | 0.01 | 0.02 |  |
| CMS18 | 6 | 0.404 | 0.425** | 0.052 | 0.01 | 0.05 | 0.01 | 0.09 |  |
| CMS32 | 8 | 0.656 | 0.580** | 0.116** | 0.01 | 0.16 | 0.01 | 0.03 |  |
| CMS13 | 13 | 0.773 | 0.732** | 0.053* | 0.01 | 0.01 | 0.01 | 0.00 |  |
| VOLP32 | 3 | 0.355 | 0.284** | 0.199** | 0.09 | 0.00 | 0.07 | 0.00 |  |
| Mean | 9.79 | 0.647 | 0.611 | 0.074 | 0.02 |  | 0.02 |  |  |
| S. D | 5.31 | 0.173 | 0.169 | 0.068 | 0.02 |  | 0.02 |  |  |

**Table S3.** Locus-by-locus genetic diversity parameters for the total sample (N = 331).

N_a_, Number of alleles. H_E_, expected heterozygosity. H_O_, observed heterozygosity. *F*_IS_, inbreeding coefficient. * P<0.05; **P<0.01. ^1^Total sample arranged in six populations. ^2^Total sample arranged in thirteen geographic regions. The monomorphic CMS17 locus is not shown.
