# Supplementary material for: Weak Genetic Structure in Northern African Dromedary Camels Reflects Their Unique Evolutionary History
Source: PLoS One. 2017 Jan 19;12(1):e0168672. doi: 10.1371/journal.pone.0168672 (PMC5245891; doi:10.1371/journal.pone.0168672)
Supplement: S5 Table — Locus-by-locus genetic diversity parameters for the Algerian (A) and the Egyptian (B) samples arranged into six different populations. (DOCX) [file pone.0168672.s005.docx]

| **A)** | **N_a_** | | | **A_r_** | | | **HWE** | | | | | | | | |
| --- | --- | --- | --- | --- | --- | --- | --- | --- | --- | --- | --- | --- | --- | --- | --- |
|  |  |  |  |  |  |  | H_e_ | H_o_ | P | H_e_ | H_o_ | P | H_e_ | H_o_ | P |
|  | AZA | RGU | TAR | AZA | RGU | TAR | AZA | | | RGU | | | TAR | | |
| **Locus ID** |  |  |  |  |  |  |  | | |  | | |  | | |
| CVRL4D | 4 | 4 | 7 | 3.4 | 3.5 | 4.0 | 0.68 | 0.65 | 0.21 | 0.62 | 0.63 | 1.00 | 0.64 | 0.56 | 0.01 |
| CVRL5D | 8 | 7 | 10 | 6.0 | 5.0 | 6.0 | 0.72 | 0.58 | 0.10 | 0.66 | 0.50 | 0.03 | 0.70 | 0.66 | 0.31 |
| CMS121 | 7 | 7 | 7 | 5.0 | 5.6 | 4.7 | 0.69 | 0.55 | 0.01 | 0.69 | 0.73 | 0.05 | 0.67 | 0.71 | 0.97 |
| CVRL6D | 3 | 3 | 3 | 3.0 | 3.0 | 2.8 | 0.43 | 0.43 | 0.21 | 0.36 | 0.34 | 0.61 | 0.33 | 0.34 | 0.25 |
| LCA66 | 4 | 6 | 6 | 3.9 | 5.2 | 5.0 | 0.68 | 0.63 | 0.54 | 0.75 | 0.75 | 0.72 | 0.74 | 0.75 | 0.28 |
| CVRL1D | 18 | 18 | 22 | 12.4 | 10.7 | 12.3 | 0.87 | 0.85 | 0.04 | 0.83 | 0.79 | 0.16 | 0.86 | 0.80 | 0.04 |
| YWLL44 | 4 | 5 | 7 | 3.7 | 4.0 | 4.0 | 0.47 | 0.45 | 0.53 | 0.51 | 0.48 | 0.01 | 0.50 | 0.48 | 0.37 |
| YWLL59 | 2 | 2 | 2 | 2.0 | 2.0 | 2.0 | 0.50 | 0.58 | 0.35 | 0.49 | 0.46 | 0.78 | 0.50 | 0.37 | 0.02 |
| CMS50 | 10 | 12 | 11 | 8.0 | 8.4 | 7.9 | 0.81 | 0.75 | 0.46 | 0.82 | 0.88 | 0.12 | 0.83 | 0.79 | 0.12 |
| CVRL8 | 2 | 2 | 2 | 2.0 | 2.0 | 2.0 | 0.37 | 0.18 | 0.00 | 0.40 | 0.41 | 1.00 | 0.36 | 0.39 | 0.38 |
| CMS9 | 9 | 9 | 10 | 7.7 | 7.0 | 8.4 | 0.86 | 0.68 | 0.00 | 0.80 | 0.56 | 0.00 | 0.83 | 0.67 | 0.00 |
| VOLP10 | 8 | 9 | 12 | 6.4 | 6.8 | 7.7 | 0.80 | 0.74 | 0.25 | 0.79 | 0.72 | 0.28 | 0.82 | 0.67 | 0.00 |
| CVRL7 | 11 | 11 | 13 | 7.9 | 7.6 | 7.9 | 0.81 | 0.78 | 0.37 | 0.82 | 0.88 | 0.54 | 0.78 | 0.76 | 0.01 |
| CMS25 | 4 | 4 | 5 | 4.0 | 4.0 | 4.0 | 0.69 | 0.75 | 0.88 | 0.68 | 0.59 | 0.14 | 0.63 | 0.63 | 0.98 |
| CMS15 | 8 | 9 | 9 | 6.5 | 6.9 | 7.3 | 0.78 | 0.75 | 0.75 | 0.76 | 0.82 | 0.20 | 0.80 | 0.71 | 0.55 |
| CMS18 | 3 | 4 | 3 | 2.6 | 3.4 | 2.9 | 0.32 | 0.30 | 0.35 | 0.37 | 0.43 | 0.67 | 0.40 | 0.46 | 0.21 |
| CMS32 | 3 | 3 | 7 | 3.0 | 3.0 | 4.5 | 0.65 | 0.53 | 0.36 | 0.63 | 0.54 | 0.22 | 0.69 | 0.64 | 0.01 |
| CMS13 | 8 | 9 | 8 | 6.4 | 6.4 | 6.8 | 0.77 | 0.68 | 0.13 | 0.74 | 0.70 | 0.84 | 0.77 | 0.76 | 0.18 |
| VOLP32 | 2 | 2 | 2 | 2.0 | 2.0 | 2.0 | 0.28 | 0.18 | 0.05 | 0.21 | 0.23 | 1.00 | 0.29 | 0.30 | 1.00 |
| Mean | 6.21 | 6.63 | 7.68 | 5.0 | 5.1 | 5.4 | 0.64 | 0.58 |  | 0.63 | 0.60 |  | 0.64 | 0.60 |  |
| S.D | 4.09 | 4.18 | 4.88 | 2.7 | 2.4 | 2.7 | 0.19 | 0.20 |  | 0.19 | 0.18 |  | 0.19 | 0.16 |  |

**Table S5.** Locus-by-locus genetic diversity parameters for the Algerian (A) and the Egyptian (B) samples arranged into six different populations.

N_a_, Number of alleles; A_r_, allelic richness; HWE, Hardy-Weinberg equilibrium; H_e_, expected heterozygosity. H_o_, observed heterozygosity. P, probability value. AZA, Azawad; RGU, Rguibi; TAR, Targui.

| **B)** | **N_a_** | | | **A_r_** | | | **HWE** | | | | | | | | |
| --- | --- | --- | --- | --- | --- | --- | --- | --- | --- | --- | --- | --- | --- | --- | --- |
|  |  |  |  |  |  |  | H_e_ | H_o_ | P | H_e_ | H_o_ | P | H_e_ | H_o_ | P |
|  | FAL | MAG | SUD | FAL | MAG | SUD | FAL | | | MAG | | | SUD | | |
| **Locus ID** |  |  |  |  |  |  |  |  |  |  |  |  |  |  |  |
| CVRL4D | 4 | 5 | 4 | 3.9 | 4.5 | 4 | 0.68 | 0.73 | 0.83 | 0.7 | 0.63 | 0.01 | 0.7 | 0.65 | 0.15 |
| CVRL5D | 6 | 7 | 5 | 5.5 | 6 | 4.6 | 0.69 | 0.57 | 0.07 | 0.63 | 0.67 | 1 | 0.55 | 0.59 | 0.79 |
| CMS121 | 7 | 10 | 6 | 6.2 | 7.8 | 5.6 | 0.73 | 0.78 | 0.72 | 0.8 | 0.77 | 0.06 | 0.69 | 0.59 | 0.32 |
| CVRL6D | 3 | 4 | 3 | 2.9 | 3 | 3 | 0.24 | 0.26 | 1 | 0.32 | 0.37 | 1 | 0.47 | 0.35 | 0.01 |
| LCA66 | 6 | 5 | 4 | 5.3 | 4.8 | 4 | 0.74 | 0.78 | 0.21 | 0.72 | 0.8 | 0.64 | 0.73 | 0.88 | 0.31 |
| CVRL1D | 12 | 16 | 12 | 11 | 11.6 | 11.7 | 0.86 | 0.7 | 0 | 0.84 | 0.9 | 0.98 | 0.86 | 0.94 | 0.58 |
| YWLL44 | 6 | 5 | 5 | 5.4 | 4 | 5 | 0.65 | 0.65 | 0.58 | 0.57 | 0.47 | 0.62 | 0.76 | 0.88 | 0.05 |
| YWLL59 | 3 | 3 | 2 | 2.9 | 2.8 | 2 | 0.36 | 0.35 | 0.04 | 0.51 | 0.47 | 0.03 | 0.43 | 0.35 | 0.57 |
| CMS50 | 8 | 9 | 9 | 7.5 | 7.7 | 8.6 | 0.85 | 0.83 | 0.05 | 0.82 | 0.9 | 0.21 | 0.85 | 0.94 | 0.14 |
| CVRL8 | 2 | 2 | 2 | 2 | 2 | 2 | 0.45 | 0.39 | 0.64 | 0.31 | 0.31 | 1 | 0.34 | 0.41 | 1 |
| CMS9 | 5 | 5 | 6 | 4.6 | 4 | 5.9 | 0.72 | 0.61 | 0.05 | 0.66 | 0.57 | 0.43 | 0.73 | 0.53 | 0.33 |
| VOLP10 | 7 | 8 | 6 | 6.8 | 6.7 | 5.9 | 0.84 | 0.74 | 0.04 | 0.8 | 0.87 | 0.41 | 0.82 | 0.65 | 0.01 |
| CVRL7 | 5 | 8 | 8 | 4.8 | 6.5 | 7.5 | 0.68 | 0.57 | 0.06 | 0.77 | 0.67 | 0.36 | 0.82 | 0.71 | 0.03 |
| CMS25 | 3 | 5 | 4 | 2.7 | 3.9 | 3.9 | 0.52 | 0.78 | 0.02 | 0.58 | 0.57 | 0.97 | 0.54 | 0.65 | 0.17 |
| CMS15 | 7 | 8 | 6 | 6.8 | 6.7 | 6 | 0.8 | 0.67 | 0.13 | 0.66 | 0.67 | 0.06 | 0.74 | 0.43 | 0.01 |
| CMS18 | 4 | 4 | 4 | 3.6 | 3.9 | 4 | 0.31 | 0.3 | 0.51 | 0.53 | 0.5 | 0.22 | 0.61 | 0.47 | 0.02 |
| CMS32 | 5 | 5 | 6 | 4.8 | 4.3 | 5.9 | 0.68 | 0.52 | 0.06 | 0.68 | 0.7 | 0.42 | 0.59 | 0.5 | 0.14 |
| CMS13 | 8 | 11 | 7 | 7.1 | 9.5 | 6.8 | 0.78 | 0.7 | 0.58 | 0.85 | 0.8 | 0.26 | 0.78 | 0.65 | 0.3 |
| VOLP32 | 3 | 3 | 2 | 3 | 2.5 | 2 | 0.64 | 0.39 | 0.01 | 0.44 | 0.23 | 0.01 | 0.49 | 0.53 | 1 |
| Mean | 5.5 | 6.5 | 5.3 | 5.1 | 5.4 | 5.2 | 0.64 | 0.6 |  | 0.64 | 0.62 |  | 0.66 | 0.62 |  |
| S.D | 2.4 | 3.4 | 2.5 | 2.2 | 2.5 | 2.4 | 0.18 | 0.18 |  | 0.16 | 0.2 |  | 0.15 | 0.19 |  |

**Table S5.** Locus-by-locus genetic diversity parameters for the Algerian (A) and the Egyptian (B) samples arranged into six different populations (Continued).

N_a_, Number of alleles; A_r_, allelic richness; HWE, Hardy-Weinberg equilibrium; H_e_, expected heterozygosity. H_o_, observed heterozygosity. P, probability value. FAL, Falahi; MAG, Maghraby; SUD, Sudani.
