# Supplementary material for: Weak Genetic Structure in Northern African Dromedary Camels Reflects Their Unique Evolutionary History
Source: PLoS One. 2017 Jan 19;12(1):e0168672. doi: 10.1371/journal.pone.0168672 (PMC5245891; doi:10.1371/journal.pone.0168672)
Supplement: S6 Table — (DOCX) [file pone.0168672.s006.docx]

|  | Al Qalaj | Iking Maryut | Birqash | Marsa Matruh | Sidi Barrany | Negeila | Bechar | Steppe | Tindouf | Adrar | Tamanrasset |
| --- | --- | --- | --- | --- | --- | --- | --- | --- | --- | --- | --- |
| Al Qalaj | 0.00000 |  |  |  |  |  |  |  |  |  |  |
| Iking Maryut | 0.02197* | 0.00000 |  |  |  |  |  |  |  |  |  |
| Birqash | 0.01452 | 0.02181* | 0.00000 |  |  |  |  |  |  |  |  |
| Marsa Matruh | 0.03168* | 0.00444 | 0.02131* | 0.00000 |  |  |  |  |  |  |  |
| Sidi Barrany | 0.02354* | 0.01057 | 0.02338* | -0.00669 | 0.00000 |  |  |  |  |  |  |
| Negeila | 0.01369 | 0.01435* | 0.01121 | 0.00839 | 0.00398 | 0.00000 |  |  |  |  |  |
| Bechar | 0.03143* | 0.01039 | 0.02751* | 0.01238 | 0.00988 | 0.02244* | 0.00000 |  |  |  |  |
| Steppe | 0.03687* | 0.02616* | 0.02577* | 0.02261* | 0.02703* | 0.03126* | 0.00152 | 0.00000 |  |  |  |
| Tindouf | 0.02608* | 0.01059* | 0.02431* | 0.00933* | 0.01341* | 0.01867* | 0.00252 | 0.00684 | 0.00000 |  |  |
| Adrar | 0.03723* | 0.02184* | 0.03102* | 0.02110* | 0.02086* | 0.02511* | 0.00594 | 0.00900* | 0.00989 | 0.00000 |  |
| Tamanrasset | 0.03069* | 0.01735* | 0.02373* | 0.01478* | 0.01561* | 0.01833* | 0.00451 | 0.01204* | 0.00449 | 0.00349 | 0.00000 |
|  |  |  |  |  |  |  |  |  |  |  |  |
| * (P<0.01) |  |  |  |  |  |  |  |  |  |  |  |

**Table S6.** Pair-wise *F*_ST_ values among the six Egyptian (Al Qalaj, Iking Maryut, Birqash, Marsa Matruh, Sidi Barrany, Negeila) and the five Algerian (Bechar, Steppe, Tindouf, Adrar, Tamanrasset) considered geographical regions.
