# Supplementary material for: Weak Genetic Structure in Northern African Dromedary Camels Reflects Their Unique Evolutionary History
Source: PLoS One. 2017 Jan 19;12(1):e0168672. doi: 10.1371/journal.pone.0168672 (PMC5245891; doi:10.1371/journal.pone.0168672)
Supplement: S7 Table — (DOCX) [file pone.0168672.s007.docx]

|  |  |  |  |  |  |  |
| --- | --- | --- | --- | --- | --- | --- |
|  | Falahy | Maghrabi | Sudany | Azawad | Rguibi | Targui |
| Falahy | 0.00000 |  |  |  |  |  |
| Maghrabi | 0.02197* | 0.00000 |  |  |  |  |
| Sudany | 0.01452 | 0.02181* | 0.00000 |  |  |  |
| Azawad | 0.03290* | 0.01759* | 0.02817* | 0.00000 |  |  |
| Rguibi | 0.03005* | 0.01337* | 0.02504* | 0.00679 | 0.00000 |  |
| Targui | 0.03105* | 0.01718* | 0.02386* | 0.00142 | 0.00561* | 0.00000 |
|  |  |  |  |  |  |  |
| * (P<0.01) |  |  |  |  |  |  |

**Table S7.** Pair-wise *F*_ST_ values among the three Egyptian (Falahy, Maghraby, Sudany) and the three Algerian (Azawad, Rguibi, Targui) populations.
