# Supplementary material for: Weak Genetic Structure in Northern African Dromedary Camels Reflects Their Unique Evolutionary History
Source: PLoS One. 2017 Jan 19;12(1):e0168672. doi: 10.1371/journal.pone.0168672 (PMC5245891; doi:10.1371/journal.pone.0168672)
Supplement: S8 Table — (DOCX) [file pone.0168672.s008.docx]

| **Country** | **Population pair** | **Unrelated** | **Half-siblings** | **Full-siblings** |
| --- | --- | --- | --- | --- |
| Algeria | A-A | 0.97 | 0.03 | 0 |
|  | T-T | 0.96 | 0.04 | 0 |
|  | R-R | 0.97 | 0.03 | 0 |
| Egypt | M-M | 0.97 | 0.03 | 0 |
|  | F-F | 0.96 | 0.04 | 0 |
|  | S-S | 0.97 | 0.03 | 0 |
| Algeria | A-T | 0.96 | 0.04 | 0 |
|  | A-R | 0.97 | 0.03 | 0 |
|  | T-R | 0.97 | 0.03 | 0 |
| Egypt | M-F | 0.97 | 0.03 | 0 |
|  | M-S | 0.98 | 0.02 | 0 |
|  | F-S | 0.97 | 0.03 | 0 |
| **-** | A-M | 0.97 | 0.03 | 0 |
| **-** | A-F | 0.96 | 0.04 | 0 |
| **-** | A-S | 0.97 | 0.03 | 0 |
| **-** | T-M | 0.97 | 0.03 | 0 |
| **-** | T-F | 0.96 | 0.04 | 0 |
| **-** | T-S | 0.96 | 0.04 | 0 |
| **-** | R-M | 0.97 | 0.03 | 0 |
| **-** | R-F | 0.97 | 0.03 | 0 |
| **-** | R-S | 0.97 | 0.03 | 0 |

**Table S8.** Proportions of pair-wise comparisons among individuals falling within the considered classes of relatedness (R).

A, Azawad; T, Targui; R, Rguibi; M, Maghraby; F, Falahi; S, Sudani. Unrelated (R < 0.25); Half-siblings (0.25 < R ≤ 0.5); Full-siblings (R > 0.5).
