# Supplementary figures and images for: Weak Genetic Structure in Northern African Dromedary Camels Reflects Their Unique Evolutionary History
Source: PLoS One. 2017 Jan 19;12(1):e0168672. doi: 10.1371/journal.pone.0168672 (PMC5245891; doi:10.1371/journal.pone.0168672)

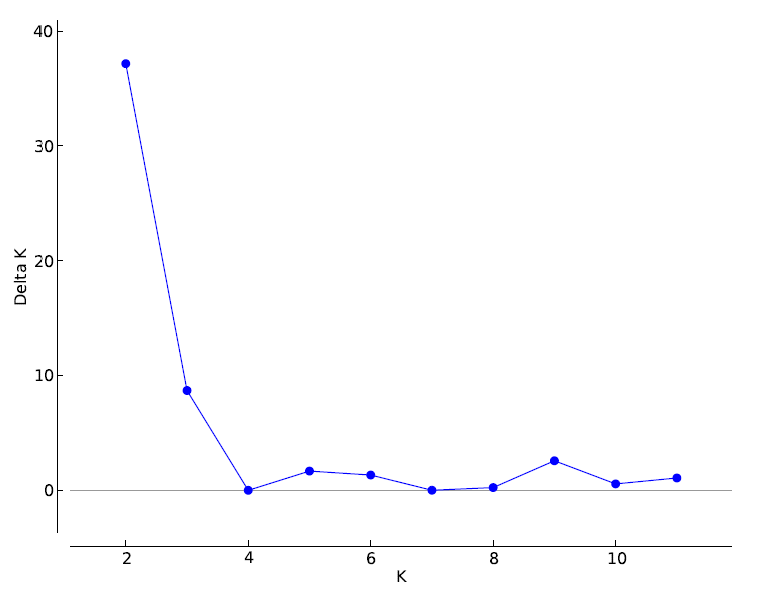


**S1 Fig.** Plot of the DeltaK statistics (Evanno *et al*., 2005) for the total sample.

Supplement: S1 Fig — (DOCX) [file pone.0168672.s009.docx]
