# Supplementary material for: Weak Genetic Structure in Northern African Dromedary Camels Reflects Their Unique Evolutionary History
Source: PLoS One. 2017 Jan 19;12(1):e0168672. doi: 10.1371/journal.pone.0168672 (PMC5245891; doi:10.1371/journal.pone.0168672)
Supplement: S2 Fig — (DOCX) [file pone.0168672.s010.docx]

**S2 Fig.** Neighbor-net network constructed using the distance of Nei (1983) considering the whole dataset arranged into three Algerian (light blue area) and three Egyptian (light yellow area) populations.
